# Predictive Factors for Adverse Event Outcomes After Transarterial Radioembolization with Yttrium-90 Resin Microspheres in Europe: Results from the Prospective Observational CIRT Study

**DOI:** 10.1007/s00270-023-03391-4

**Published:** 2023-03-13

**Authors:** Geert Maleux, Thomas Albrecht, Dirk Arnold, Irene Bargellini, Roberto Cianni, Thomas Helmberger, Frank Kolligs, Graham Munneke, Bora Peynircioglu, Bruno Sangro, Niklaus Schaefer, Helena Pereira, Bleranda Zeka, Niels de Jong, José I. Bilbao, the CIRT Principal Investigators, Thomas Albrecht, Olivier D’Archambeau, Tugsan Balli, Sadik Bilgic, Allan Bloom, Roberto Cioni, Roman Fischbach, Patrick Flamen, Laurent Gerard, Rita Golfieri, Gerd Grözinger, Marcus Katoh, Michael Koehler, Jan Robert Kröger, Christiane Kuhl, Franco Orsi, Murat Özgün, Peter Reimer, Maxime Ronot, Axel Schmid, Alessandro Vit, Geert Maleux, Bruno Sangro, Maciej Pech, Thomas Helmberger, Roberto Cianni, Thomas Pfammatter

**Affiliations:** 1 Radiology, Universitair Ziekenhuis Leuven, Herestraat 49, 3000 Leuven, Belgium; 2 Department for Radiology and Interventional Therapy, Vivantes Klinikum Neukölln, Rudower Str. 48, 12351 Berlin, Germany; 3 Oncology and Hematology, Asklepios Tumorzentrum Hamburg, AK Altona, Paul-Ehrlich-Str. 1, 22763 Hamburg, Germany; 4 Department of Vascular and Interventional Radiology, Careggi University Hospital, Largo Brambilla 3, 50134 Florence, Italy; 5 Department of Interventional Radiology, S. Camillo Hospital, Circonvallazione Gianicolense, 85, 00149 Rome, Italy; 6 Department of Radiology, Neuroradiology and Minimal-Invasive Therapy, Klinikum Bogenhausen, Englschalkinger Str. 77, 81925 Munich, Germany; 7 Department of Internal Medicine and Gastroenterology, Helios Klinikum Berlin-Buch, Schwanebecker Chaussee 50, 13125 Berlin, Germany; 8 Interventional Oncology, University College London Hospitals NHS Foundation Trust, 250 Euston Road, London, NW1 2PG UK; 9 Department of Radiology, School of Medicine, Hacettepe University, Sihhiye Campus, 06100 Ankara, Turkey; 10 Liver Unit and HPB Oncology Area, Clínica Universidad de Navarra and CIBEREHD, Avda. Pio XII 36, 31008 Pamplona, Spain; 11 Service de Médecine Nucléaire et Imagerie Moléculaire, University Hospital of Lausanne (CHUV), Rue du Bugnon 46, 1011 Lausanne, Switzerland; 12 Assistance Publique-Hôpitaux de Paris, Hôpital Européen Georges-Pompidou, Unité de Recherche Clinique, Paris, France; 13 Centre d’Investigation Clinique 1418 (CIC1418), INSERM, Paris, France; 14 Clinical Research Department, Cardiovascular and Interventional Radiological Society of Europe, Neutorgasse 9, 1010 Vienna, Austria; 15 Interventional Radiology, Clínica Universidad de Navarra, Avenida Pio XII, No 36, 31008 Pamplona, Spain

## Abstract

**Background:**

Using data collected in the prospective observational study CIRSE Registry for SIR-Spheres Therapy, the present study aimed at identifying predictors of adverse events (AEs) following transarterial radioembolization (TARE) with Yttrium-90 resin microspheres for liver tumours.

**Methods:**

We analysed 1027 patients enrolled between January 2015 and December 2017 and followed up for 24 months. Four hundred and twenty-two patients with hepatocellular carcinoma (HCC), 120 with intrahepatic carcinoma (ICC), 237 with colorectal liver metastases and 248 with liver metastases from other primaries were included. Prognostic factors were calculated with a univariable analysis by using the overall AEs burden score (AEBS).

**Results:**

All-cause AEs were reported in 401/1027 (39.1%) patients, with AEs associated with TARE, such as abdominal pain (16.6%), fatigue (17%), and nausea (11.7%) reported most frequently. Grade 3 or higher AEs were reported in 92/1027 (9%) patients. Reports on grade ≥ 3 gastrointestinal ulcerations (0.4%), gastritis (0.3%), radiation cholecystitis (0.2%) or radioembolization-induced liver disease (0.5%) were uncommon. Univariable analysis showed that in HCC, AEBS increased for Eastern Cooperative Oncology Group (ECOG) 0 (*p* = 0.0045), 1 tumour nodule (0.0081),  > 1 TARE treatment (*p* = 0.0224), no prophylactic embolization (*p* = 0.0211), partition model dosimetry (*p* = 0.0007) and unilobar treatment target (0.0032). For ICC,  > 1 TARE treatment was associated with an increase in AEBS (*p* = 0.0224), and for colorectal liver metastases, ECOG 0 (*p* = 0.0188), > 2 prior systemic treatments (*p* = 0.0127), and 1 tumour nodule (*p* = 0.0155) were associated with an increased AEBS.

**Conclusion:**

Our study confirms that TARE is a safe treatment with low toxicity and a minimal impact on quality of life.

**Supplementary Information:**

The online version contains supplementary material available at 10.1007/s00270-023-03391-4.

## Introduction

Transarterial radioembolization (TARE) is a treatment modality for cancer patients with liver-dominant disease not suitable for surgical or ablative therapies, or who experienced no response, significant side effects or intolerance when treated with systemic therapies [1–9]. Depending on the origin of the tumour—be it primary such as hepatocellular carcinoma (HCC), intrahepatic cholangiocarcinoma (ICC) or metastatic liver disease—TARE is included as a palliative treatment option in several treatment guidelines, due to its favourable toxicity profile and ability to facilitate local tumour control [1–10].

Previous reports on the safety and toxicity after TARE follow a trend of focusing on grade ≥ 3 adverse events (AEs) [11–15]. While insightful, those reports ignore the more commonly occurring grade 1 and 2 AEs. Despite their mildness, frequently occurring grade 1 and 2 AEs can put a burden on patients’ health and should, therefore, be included in the safety evaluation of a treatment. To that end, La-Rademacher et al. (2020) developed a single measure that reflects the overall AE burden by including all AEs graded and reported during a trial into a single numeric value [16]. This adverse event burden score (AEBS) allows for inter- and intra-study comparisons between groups and treatments and can be applied to find risk factors for adverse events or associations between adverse events and other numeric factors, such as quality-of-life data.

Using the data from the prospective real-world observational study, the CIRSE Registry for SIR-Spheres Therapy (CIRT), we aimed to provide a comprehensive overview of adverse events observed after TARE and to find factors in the patient baseline data and treatment modalities that can predict an increased AEBS or reduced global health score (GHS). This evaluation was performed for the whole cohort, with an emphasis on HCC, ICC and colorectal liver metastases (mCRC).

## Methods

### Study Design

The prospective multicentre observational study CIRT (NCT02305459) was conducted by the Cardiovascular and Interventional Radiological Society of Europe (CIRSE) to evaluate the real-world clinical application and outcomes of TARE with Y90 resin microspheres (SIR-Spheres® Y-90 resin microspheres, Sirtex Medical Pty Limited; St. Leonards, NSW, Australia). Sites were invited to participate if they performed at least 40 TARE cases in total and ten cases in the twelve months prior to invitation. The 27 participating sites were identified and enrolled between April 2014 and April 2017.

The CIRT methodology was previously published [17]. Data were collected using a customised electronic data capturing system and electronic case report form that was developed by ConexSys Inc (Lincoln, RI, USA) and hosted on a local secure server in Vienna, Austria, maintained by ITEA (Vienna, Austria). Statistical analyses were performed in SAS 9.4 (SAS Institute, Cary, NC, USA) and RStudio under R4.0.0 (R Foundation, Vienna, Austria).

### Patient Selection and Data Collection

Patients included in the analysis were adults scheduled to receive TARE with Y90 resin microspheres for primary or metastatic liver cancer. There were no specific inclusion or exclusion criteria. The indication for TARE, the treatment design, the methods used for dose calculation and the follow-up regime were made according to the centres’ internal standards. All included patients signed an informed consent form. This research project was performed in accordance with the ethical standards of the applicable institutional and/or national ethics committees and with the 1964 Helsinki Declaration and its later amendments or comparable ethical standards.

At the time of the first treatment, baseline data, demographics and treatment-related data were collected. Information concerning safety, toxicities and quality of life were gathered at every follow-up up to 24 months. Safety outcomes are described as severe day-of-treatment complications and occurrences of any adverse events after treatment, according to the Common Terminology Criteria for Adverse Events, version 4.03. Previous studies have associated adverse events such as abdominal pain, fatigue, fever, nausea, vomiting, gastrointestinal ulceration, gastritis, radiation cholecystitis, radiation pancreatitis, radiation pneumonitis and radioembolization-induced liver disease (REILD) with the application of TARE and were, thus, specifically included in the case report form [18–20]. An open text field allowed for collecting details on occurrences of other adverse events. Quality-of-life data were collected on a voluntary basis for the patient using the questionnaire QLQ-C30 from the European Organisation for the Research and Treatment of Cancer (EORTC). The questionnaire was presented before the TARE treatment and at every follow-up. Remote monitoring was performed, but no onsite monitoring or source document verification was done.

### Definitions and Outcome Measures

To describe and compare safety data, the proportion of patients with at least 1 AE, as well as the AE burden score (AEBS), were used. To summarise, the AEBS represents the weighted sum of all AEs and their respective weighted grades [16]. Since all AEs were graded according to the Common Terminology Criteria for Adverse Events (CTCAE), which reflects comparable severity between AE types, the weight of an AE type was defined as the CTCAE grade. Therefore, the AEBS was calculated by taking the sum of all AE grades per patient (see Supplement 1 for an extended explanation of the AEBS, including the weight of grade 5 AEs, and AEs excluded from the AEBS calculation).

Deterioration of quality-of-life (QOL) data was evaluated using the EORTC Scoring Manual 3.0 [21]. A 10-point change in the score from baseline (first TARE treatment) was considered to be clinically relevant. If there was a 10-point decrease in the function domain or a 10-point increase in the symptom score from baseline, the patient’s QOL in that domain or for that symptom was considered to be deteriorated. This evaluation could be done only for patients who completed the QOL questionnaire at baseline and during, at least, one follow-up visit. Here, we report only on GHS.

### Statistics

Data are presented as mean ± standard deviation or median (interquartile range [IQR]) for continuous variables and number (%) for categorical variables. Adverse events are reported as occurrences per patient. When reported per time interval, adverse events are reported per occurrence within the time intervals < 1 month, 1–4 months, and > 4 months. Data are presented for the whole cohort and cancer types with > 100 patients (HCC, ICC and mCRC).

Univariable analyses were performed with the Wilcoxon rank-sum test if the number of groups was 2, or with the Kruskal–Wallis test (nonparametric alternative to the ANOVA) if the number of groups exceeded 2, due to the non-normal distribution of the AEBS data. The statistical significance level was set to *p* < 0.05 (two-sided). Due to the large number of patients for which no AE was reported (AEBS = 0), it was not possible to perform a multivariable analysis.

## Results

We analysed 1197 TARE treatments from 1027 patients included in the CIRT study. Data on baseline and treatment application have been previously reported, which also included, as supplementary information, AE data outlined in Table 1 [22]. Safety data in patients with mCRC and HCC have been previously reported as supplementary information [23, 24].

**Table 1:** Safety outcomes after TARE

Variables	Categories	HCC	ICC	mCRC	Other mets	All
Length of hospital stay	*n*	496 (100)	144 (100)	264 (100)	293 (100)	1197 (100)
	< 24 h	116 (23.4)	27 (18.8)	57 (21.6)	85 (29.0)	285 (23.8)
	24 to < 36 h	64 (12.9)	12 (8.3)	41 (15.5)	37 (12.6)	154 (12.9)
	36 to < 48 h	52 (10.5)	12 (8.3)	39 (14.8)	44 (15.0)	147 (12.3)
	48 to < 72 h	221 (44.6)	74 (51.4)	79 (29.9)	91 (31.0)	465 (38.8)
	≥ 72 h	43 (8.7)	19 (13.2)	48 (18.2)	36 (12.3)	146 (12.2)
Deceased	*n*	422 (100)	120 (100)	237 (100)	248 (100)	1027 (100)
	Within 30 days*	3 (0.7)	1 (0.8)	4 (1.7)	2 (0.8)	10 (1.0)
Severe periprocedural complications	*n*	11 (2.2)	6 (4.2)	9 (3.4)	19 (6.5)	44 (3.7)
	Abdominal pain	3 (0.6)	3 (2.1)	8 (3.0)	12 (4.8)	26 (2.3)
	Vomiting	2 (0.4)	1 (0.7)	3 (1.1)	2 (0.7)	8 (0.7)
	Other	6 (1.2)	2 (1.4)	1 (0.4)	3 (1)	10 (0.8)
Patients with at least one adverse event		155 (36.7)	49 (40.8)	96 (40.5)	102 (41.1)	402 (39.1)
Patients with at least one adverse events (all)	Abdominal Pain	60 (14.2)	25 (20.8)	34 (14.3)	51 (20.5)	170 (16.6)
	Fatigue	65 (15.4)	23 (19.2)	33 (13.9)	54 (21.7)	175 (17.0)
	Fever	25 (5.9)	7 (5.8)	14 (5.9)	9 (3.6)	55 (5.4)
	Nausea	37 (8.8)	14 (11.7)	28 (11.8)	41 (16.5)	120 (11.7)
	Vomiting	22 (5.2)	9 (7.5)	15 (6.3)	20 (8.1)	66 (6.4)
	Gastrointestinal Ulceration	3 (0.7)	5 (4.2)	3 (1.3)	2 (0.8)	13 (1.3)
	Gastritis	3 (0.7)	2 (1.7)	6 (2.5)	3 (1.2)	14 (1.4)
	Radiation Cholecystitis	0 (0.0)	1 (0.8)	1 (0.4)	2 (0.8)	4 (0.4)
	Radiation Pancreatitis	0 (0.0)	0 (0.0)	0 (0.0)	1 (0.4)	1 (0.1)
	Radiation pneumonitis	0 (0.0)	0 (0.0)	0 (0.0)	0 (0.0)	0 (0.0)
	Radioembolization-Induced Liver Disease	6 (1.4)	3 (2.5)	1 (0.4)	3 (1.2)	13 (1.3)
	Other	90 (21.3)	25 (20.8)	63 (26.6)	48 (22.6)	234 (22.8)
Patients with at least one grade ≥ 3 adverse event		30 (7.1)	15 (12.5)	25 (10.5)	22 (8.9)	92 (9.0)
Patients with at least one ≥ grade 3 adverse events	Abdominal Pain	9 (2.1)	4 (3.3)	4 (1.7)	8 (3.2)	25 (2.4)
	Fatigue	6 (1.4)	2 (1.7)	0 (0.0)	6 (2.4)	14 (1.4)
	Fever	2 (0.5)	0 (0.0)	0 (0.0)	0 (0.0)	2 (0.2)
	Nausea	3 (0.7)	0 (0.0)	1 (0.4)	1 (0.4)	5 (0.5)
	Vomiting	2 (0.5)	0 (0.0)	0 (0.0)	0 (0.0)	2 (0.2)
	Gastrointestinal Ulceration	1 (0.2)	1 (0.8)	2 (0.8)	0 (0.0)	4 (0.4)
	Gastritis	0 (0.0)	1 (0.8)	2 (0.8)	0 (0.0)	3 (0.3)
	Radiation Cholecystitis	0 (0.0)	1 (0.8)	1 (0.4)	0 (0.0)	2 (0.2)
	Radiation Pancreatitis	0 (0.0)	0 (0.0)	0 (0.0)	0 (0.0)	0 (0.0)
	Radiation pneumonitis	0 (0.0)	0 (0.0)	0 (0.0)	0 (0.0)	0 (0.0)
	Radioembolization-Induced Liver Disease	3 (0.7)**	2 (1.7)	0 (0.0)	0 (0.0)	5 (0.5)
	Other	15 (3.6)	8 (6.7)	18 (7.6)	10 (4.0)	51 (5.0)

Data are presented as *n* (%)

*ICC* intrahepatic cholangiocarcinoma, *HCC* hepatocellular carcinoma, *mCRC* metastatic colorectal cancer, *TARE* transarterial radioembolization

The study included 1027 patients with a total of 1197 treatments

*7/10 patients died from intra-hepatic or extra-hepatic disease progression; 1 patient died from pleural effusion and ascites; for 2 patients, the cause of death is unrelated to the treatment or the disease

**2/3 (66.7%) cases of Radioembolization-Induced Liver Disease in HCC patients were grade 5

### Safety

Across indications, the number of patients who experienced at least one severe periprocedural complication after a treatment was low (44/1197, 3.7%), primarily abdominal pain (26/1197, 2.3%, Table 1). All-cause AEs were reported in 402/1027 (39.1%) patients, and grade 3 or higher AEs were reported in 92/1027 (9%) patients. Patients with other grade ≥ 3 AEs (51/1027, 5.0%) are reported in Table 2. Within 1 month after treatment (Table 3), the largest groups of reported AEs were abdominal pain (126/909, 13.9%), fatigue (104/909, 11.4%) and nausea (69/909, 7.6%). Between 1 and 4 months, occurrences of these AEs decreased, while cases of radiation cholecystitis (3/726, 0.4%), radiation pancreatitis (1/726, 0.1%), REILD (10/726, 1.4%) and other AEs (62/726, 8.5%%) increased. AEs in the category “Other” occurred mostly after 4 months (156/639, 24.4%). Supplement 2 provides the occurrences of AEs per time interval for HCC, ICC and mCRC. The thirty-day mortality rate was 10/1027 (1.0%, Table 1). Abnormal laboratory values (Table 4) were reported in 792/1027 (77.1%) patients, with 245/1027 (23.9%) patients showing at least 1 value with grade 3 or higher.

**Table 2:** Other serious adverse events

Category*	Adverse event	Grade 3 (*n* = 72)	Grade 4 (*n* = 10)	Grade 5 (*n* = 3)	Total (*n* = 84)
Analytical	Anaemia	3			3
	Thrombocytopenia	1			1
Bleeding	Oesophagus	4	1		4
	Gastrointestinal	1	2	1	4
	Haemorrhage at ileostomy	1			1
	Fundal variceal haemorrhage	1			1
	Bleeding from gastric ulcer	1			1
	Left liver lobe	1			1
	Rectum	1			1
Cardio-pulmonary	Heart failure	1			1
	Respiratory failure		1		1
	Pleural effusion	1	1		2
	Circulatory insufficiency	1			1
	Central embolism of the pulmonal artery	1			1
	Dyspnoea	1			1
Digestive	Sub-occlusion	1	1		2
	Diarrhoea	3	1		4
	Mouth ulceration	1			1
General	Depression	2			2
	Somnolence	4			4
	Anorexia	3			3
	Weight loss	1			1
	Sleep disorder	1			1
Infectious	Cholangio-sepsis	1			1
	Streptococci infection	1			1
	Sepsis for staphylococcus aureus with endocarditis	1			1
	Bilateral lower limb gangrene	1			1
	Infection of necrotic tumour areas in liver	1			1
	Infection with E. coli	1			1
	Infected biloma	1			1
	Spontaneous bacterial peritonitis	1			1
Liver and portal system	Cholangitis	2	1		3
	Ascites	7	1		8
	Jaundice	3	1		4
	Cholestasis	1			1
	Intrahepatic thrombosis of the portal vein	1			1
	Infarction of ventral part of the spleen	1			1
	Hepatic encephalopathy	2			2
	Biloma	1			1
	Deterioration of the liver function	1			1
Neurological, pain, and other sensitive disorders	Hand-foot syndrome	3			3
	Bone pain	2			2
	Epileptic seizure	1			1
	Back pain	1			1
Renal and fluid balance	Acute renal failure			2	2
Other	Wound rupture after liver surgery	1			1
	Teeth destroyed	1			1
	Rash of the skin due to antibiotics	1			1

*51 patients experienced 84 severe adverse events in the “other” category

**Table 3:** Patients with at least 1 adverse event per time interval: < 1 month, 1–4 months, > 4 months

	< 1 month	1–4 months	> 4 months
*Patients with at least 1 follow-up per time interval*	*909*	*726*	*639*
*Adverse events (all grades, n)*	*392*	*302*	*472*
Abdominal pain	126 (13.9)	65 (9)	94 (14.7)
Fatigue	104 (11.4)	75 (10.3)	128 (20)
Fever	11 (1.2)	19 (2.6)	20 (3.1)
Nausea	69 (7.6)	44 (6.1)	50 (7.8)
Vomiting	48 (5.4)	13 (1.8)	16 (2.5)
Gastrointestinal ulceration	3 (0.3)	6 (0.8)	5 (0.8)
Gastritis	8 (0.9)	5 (0.7)	1 (0.2)
Radiation cholecystitis	0 (0)	3 (0.4)	1 (0.2)
Radiation pancreatitis	0 (0)	1 (0.1)	0 (0)
Radiation pneumonitis	0 (0)	0 (0)	0 (0)
Radioembolization-induced liver disease	2 (0.2)	10 (1.4)	1 (0.2)
Other	20 (2.2)	62 (8.5)	156 (24.4)
*Adverse events (grade 3–5, n)*	*52*	*40*	*67*
Abdominal pain	36 (4)	6 (0.8)	11 (1.7)
Fatigue	3 (0.3)	5 (0.7)	7 (1.1)
Fever	0 (0)	1 (0.1)	1 (0.2)
Nausea	0 (0)	4 (0.6)	1 (0.2)
Vomiting	8 (0.9)	1 (0.1)	1 (0.2)
Gastrointestinal ulceration	0 (0)	3 (0.4)	2 (0.3)
Gastritis	2 (0.2)	1 (0.1)	0 (0)
Radiation cholecystitis	0 (0)	0 (0)	0 (0)
Radiation pancreatitis	0 (0)	0 (0)	0 (0)
Radiation pneumonitis	0 (0)	0 (0)	0 (0)
Radioembolization-induced liver disease	1 (0.1)	4 (0.6)	0 (0)
Other	2 (0.2)	15 (2.1)	44 (6.9)

Data are presented as *n* (%). The percentage is taken over the number of patients with at least 1 follow-up per time interval

The number of adverse events per time intervals for hepatocellular carcinoma, intrahepatic cholangiocarcinoma and colorectal cancer liver metastases are included in Supplement 2

**Table 4:** Abnormal laboratory values: patients with at least one occurrence per grade

Category	HCC	ICC	mCRC	Other mets (248)	Total (1027)
Patients with at least 1 abnormal laboratory value	359 (85.1)	96 (80)	158 (66.7)	179 (72.2)	792 (77.1)
Patients with at least 1 grade ≥ 3 abnormal laboratory value	129 (30.6)	27 (22.5)	42 (17.7)	47 (19)	245 (23.9)
*Albumin decreased—grade*					
n	422	120	237	248	1027
1	167 (39.6)	26 (21.7)	36 (15.2)	47 (19)	276 (26.9)
2	117 (27.7)	25 (20.8)	29 (12.2)	38 (15.3)	209 (20.4)
3	19 (4.5)	4 (3.3)	9 (3.8)	2 (0.8)	34 (3.3)
*ALT increased—grade*					
n	422	120	237	248	1027
1	197 (46.7)	49 (40.8)	80 (33.8)	106 (42.7)	432 (42.1)
2	31 (7.3)	2 (1.7)	15 (6.3)	20 (8.1)	68 (6.6)
3	13 (3.1)	0 (0)	9 (3.8)	6 (2.4)	28 (2.7)
4	0 (0)	0 (0)	0 (0)	1 (0.4)	1 (0.1)
*AST increased—grade*					
n	422	120	237	248	1027
1	258 (61.1)	55 (45.8)	108 (45.6)	118 (47.6)	539 (52.5)
2	66 (15.6)	12 (10)	24 (10.1)	23 (9.3)	125 (12.2)
3	25 (5.9)	3 (2.5)	13 (5.5)	14 (5.6)	55 (5.4)
4	3 (0.7)	0 (0)	0 (0)	1 (0.4)	4 (0.4)
*Bilirubin increased—grade*					
n	422	120	237	248	1027
1	154 (36.5)	24 (20)	57 (24.1)	44 (17.7)	279 (27.2)
2	141 (33.4)	22 (18.3)	35 (14.8)	34 (13.7)	232 (22.6)
3	63 (14.9)	15 (12.5)	19 (8)	20 (8.1)	117 (11.4)
4	18 (4.3)	3 (2.5)	6 (2.5)	7 (2.8)	34 (3.3)
*INR increased*					
n	422	120	237	248	1027
1	133 (31.5)	17 (14.2)	38 (16)	26 (10.5)	214 (20.8)
2	57 (13.5)	10 (8.3)	16 (6.8)	14 (5.6)	97 (9.4)
3	14 (3.3)	1 (0.8)	1 (0.4)	3 (1.2)	19 (1.9)
*Neutrophil count decreased—grade*					
n	422	120	237	248	1027
1	36 (8.5)	14 (11.7)	8 (3.4)	16 (6.5)	74 (7.2)
2	22 (5.2)	5 (4.2)	7 (3)	8 (3.2)	42 (4.1)
3	6 (1.4)	1 (0.8)	5 (2.1)	11 (4.4)	23 (2.2)
4	0 (0)	1 (0.8)	1 (0.4)	1 (0.4)	3 (0.3)
*Platelet count decreased—grade*					
n	422	120	237	248	1027
1	216 (51.2)	55 (45.8)	58 (24.5)	78 (31.5)	407 (39.6)
2	73 (17.3)	17 (14.2)	10 (4.2)	10 (4)	110 (10.7)
3	27 (6.4)	5 (4.2)	1 (0.4)	5 (2)	38 (3.7)
4	5 (1.2)	1 (0.8)	2 (0.8)	1 (0.4)	9 (0.9)

Data are presented as *n* (%)

*ALT* Alanine transaminase, *AST* Aspartate aminotransferase, *ICC* intrahepatic cholangiocarcinoma, *INR* International Normalised Ratio, *HCC* hepatocellular carcinoma, *mCRC* metastatic colorectal cancer, *TARE* transarterial radioembolization

### Prognostic Factors

For the calculation of the overall AEBS, 620 patients had no AEs (AEBS = 0), 31 patients with ungraded AEs and 118 patients AEs only in the “Other” category were excluded, leaving a total of 258 patients with an AEBS > 0. The mean and standard deviation for the total overall AEBS were 1.4 ± 3.8 for all AEs and 0.2 ± 1.2 for serious AEs. Univariable analysis (Table 5) showed that the variables > 1 TARE treatments (mean ± SD 2.6 ± 5.2, *p* < 0.0001), no prophylactic embolization (2.0 ± 4.9, *p* = 0.0222), partition model (2.1 ± 4.9, *p* = 0.022), > 2 prior systemic treatments (2.1 ± 5.0, *p* < 0.0001) and unilobar treatments (2.2 ± 4.6 for left lobe treatments and 1.7 ± 4.2 for right lobe treatments, *p* = 0.0448) were predictors of an increased overall AEBS, in addition to Eastern Cooperative Oncology Group (ECOG) 0 (1.9 ± 4.5, *p* < 0.0001), 1 tumour nodule (2.5 ± 5.4, *p* = 0.0002) and the percentage of tumour invasion in the left liver lobe of > 20% (3.5 ± 5.5, *p* = 0.0283). Furthermore, cancer type was associated with a difference in AEBS (*p* = 0.0422).

**Table 5:** Univariable analysis of statistically significant predictors of increased overall adverse events burden score

Variable	Categories	N	Mean AEBS ± SD	*P* value
*Complete cohort (all cancer types, n* = *1027)*				
Cancer type	Hepatocellular carcinoma	422	1.2 ± 3.8	0.0422
	Intrahepatic cholangiocarcinoma	120	2.0 ± 4.7	
	Colorectal cancer liver mets	237	1.2 ± 3.1	
	Neuroendocrine tumour liver mets	58	2.3 ± 5.5	
	Breast cancer liver mets	47	2.2 ± 4.7	
	Pancreatic cancer liver mets	32	1.1 ± 1.5	
	Melanoma liver mets	32	1.2 ± 1.8	
ECOG	0	625	1.9 ± 4.5	< 0.0001
	1	316	0.7 ± 1.8	
	≥ 2	75	0.4 ± 1.4	
Number of prior treatments	0	521	1.3 ± 3.9	< 0.0001
	1	191	0.5 ± 1.4	
	2	98	0.8 ± 2.0	
	> 2	217	2.6 ± 5.0	
Number of tumour nodules	1	230	2.5 ± 5.4	0.0002
	2–5	253	1.2 ± 3.4	
	> 5	245	1.3 ± 3.3	
	Uncountable	299	0.8 ± 2.5	
	10–20%	99	2.7 ± 6.1	
	> 20%	103	2.2 ± 4.4	
Percentage tumour invasion in the liver (left lobe)	< 10%	224	2.1 ± 4.9	0.0283
	10–20%	73	2.2 ± 4.9	
	> 20%	85	3.5 ± 5.5	
Number of TARE treatments	1	869	1.2 ± 3.4	< .0001
	≥ 2	158	2.6 ± 5.2	
Prophylactic embolization	Yes	412	0.8 ± 2.1	0.0222
	No	477	2.0 ± 4.9	
Dose methodology	BSA/mBSA	736	1.1 ± 3.2	0.0022
	Partition	279	2.1 ± 4.9	
TARE Target	Left lobe	124	2.2 ± 4.6	0.0448
	Right lobe	337	1.7 ± 4.2	
	Whole liver (segmental)	98	0.9 ± 3.3	
	Whole liver (sequential)	136	1.0 ± 2.4	
	Whole liver (single catheter)	121	1.1 ± 3.7	
	Whole liver (split administration)	171	1.1 ± 2.9	
*Hepatocellular carcinoma (n* = *422)*				
ECOG	0	260	1.7 ± 4.7	0.0045
	1	131	0.6 ± 1.5	
	≥ 2	31	0.0 ± 0.1	
Number of prior treatments	0	381	1.3 ± 3.9	0.0107
	1	35	0.6 ± 2.2	
	2	4	1.0 ± 2.0	
	> 2	2	8.5 ± 0.7	
Number of tumour nodules	1	136	2.2 ± 5.0	0.0081
	2–5	138	1.0 ± 3.5	
	> 5	72	1.0 ± 3.4	
	Uncountable	76	0.3 ± 1.1	
Number of TARE treatments	1	353	1.2 ± 3.8	0.0224
	≥ 2	69	1.6 ± 3.9	
Prophylactic embolization	No	217	1.8 ± 4.9	0.0211
	Yes	138	0.7 ± 2.2	
Dose methodology	BSA/mBSA	245	0.7 ± 2.8	0.0211
	Partition	173	2.0 ± 4.8	
TARE Target	Left lobe	56	1.8 ± 4.6	0.0032
	Right lobe	177	1.6 ± 4.2	
	Whole liver (segmental)	66	0.2 ± 0.9	
	Whole liver (sequential)	33	1.1 ± 2.8	
	Whole liver (single catheter)	53	1.4 ± 5.1	
	Whole liver (split administration)	37	0.2 ± 0.9	
*Intrahepatic cholangiocarcinoma (n* = *120)*				
Number of TARE treatments	1	97	1.6 ± 3.5	0.0224
	≥ 2	23	3.7 ± 7.8	
*Colorectal liver metastases (n* = *237)*				
ECOG	0	143	1.8 ± 3.8	0.0188
	1	72	0.5 ± 1.5	
	≥ 2	18	0.5 ± 1.1	
Number of prior treatments	0	21	1.1 ± 2.6	0.0127
	1	69	0.3 ± 1.0	
	2	41	0.7 ± 2.1	
	> 2	106	2.1 ± 4.1	
Number of tumour nodules	1	27	2.5 ± 4.8	0.0155
	2–5	50	1.7 ± 3.5	
	> 5	79	1.4 ± 3.2	
	Uncountable	81	0.4 ± 1.4	

Other, non-significant variables for the whole cohort, hepatocellular carcinoma, intrahepatic cholangiocarcinoma and colorectal liver metastases included in the univariable analysis: Gender; Presence of extra-hepatic disease; prior systemic therapy; prior hepatic procedures; percentage of tumour invasion in the whole liver; percentage of tumour invasion in the right liver lobe; Unilobar treatment, left vs right; Prescribed activity, < 1 GBq, 1.1–1.15 GBq, 1.5–1.82 GBq, > 1.82 GBq. The complete tables can be found in Supplement 3

Analyses were performed using the Wilcoxon rank-sum test if the number of groups is 2 or with the Kruskal–Wallis test (nonparametric alternative to the ANOVA) if the number of groups exceed 2. Multivariable analysis could not be performed due to the high number of patients with no adverse events

*BSA* body surface area, *ECOG* Eastern Cooperative Oncology Group, *GBq* giga-becquerel, *mBSA* modified body surface area, *SD* standard deviation, *TARE* transarterial radioembolization

Variables associated with a difference in AEBS for HCC were ECOG 0 (*p* = 0.0045), number of prior systemic treatments (*p* = 0.0107), 1 tumour nodule (0.0081), number of TARE treatments (*p* = 0.0224), no prophylactic embolization (*p* = 0.0211) partition model dosimetry (*p* = 0.0007) and unilobar treatment target (0.0032). For ICC, only the number of TARE treatments was associated with a change in AEBS (*p* = 0.0224), and for colorectal liver metastases, ECOG 0 (*p* = 0.0188), > 2 prior systemic treatments (*p* = 0.0127), and 1 tumour nodule (*p* = 0.0155) were associated with a change in AEBS (Table 5). Supplements 3 A-D describe the outcomes of all variables included in the univariable analysis.

To further investigate the results of the univariable analysis, a comparison of reported AEs per time interval (< 1 month, 1–4 months, and > 4 months) was performed (Supplement 4A–E). This comparison revealed that the reporting of symptomatic adverse events (abdominal pain, fatigue, nausea, vomiting and fever) was different between patients with ECOG 0 compared to ECOG > 0, 1 tumour nodule compared to > 1 tumour nodule, unilobar treatments versus bilobar treatments, activity calculations with partition model compared to standard body surface area, and patients that had no prophylactic embolization compared to those that had prophylactic embolization.

### Global Health Score

**Fig. 1:**
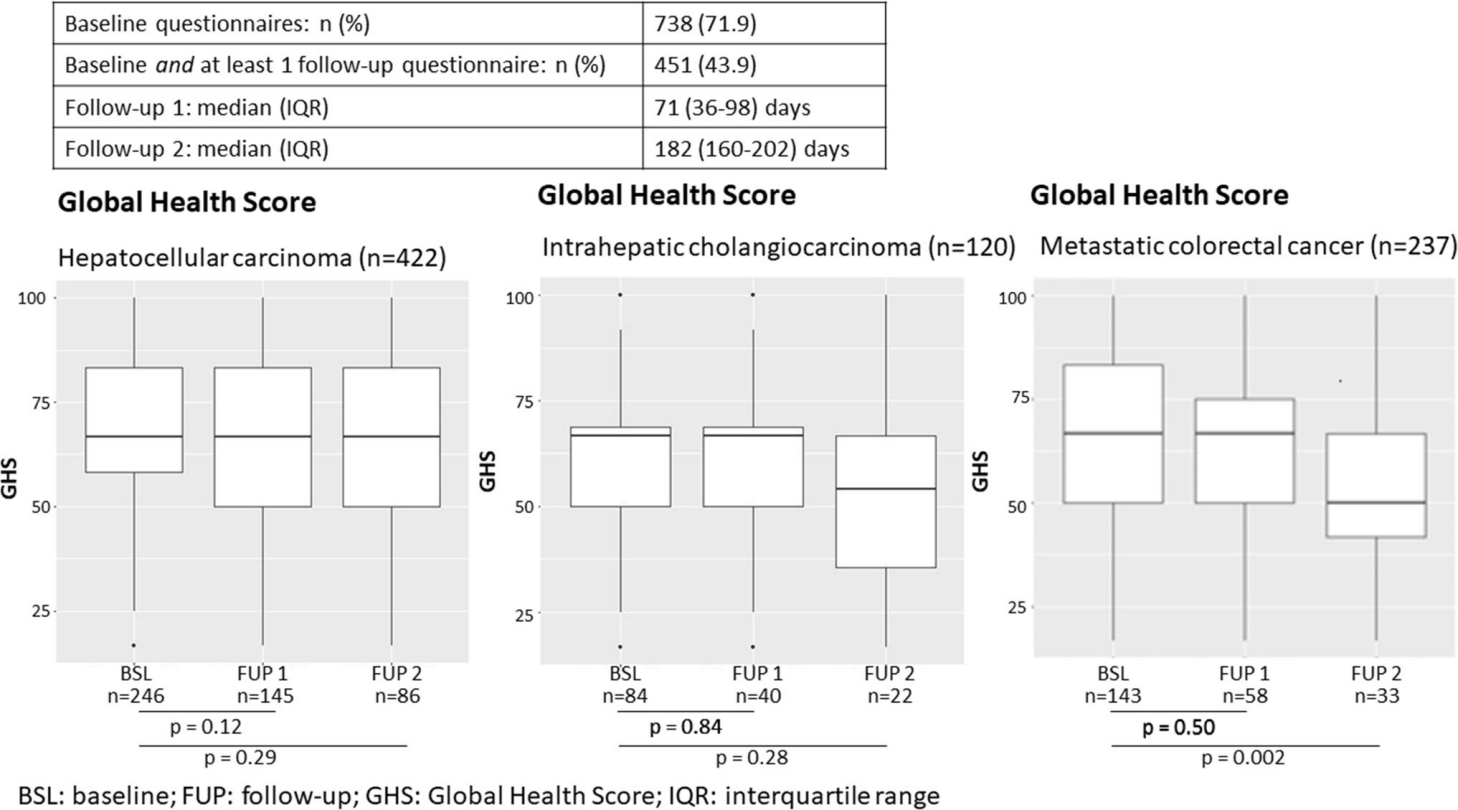
Change in Global Health Score from baseline to follow-up 1 and follow-up 2 for HCC, ICC and mCRC

In total, 451 patients were eligible for quality-of-life deterioration evaluation, having completed a questionnaire both at baseline and during at least one follow-up visit. Deterioration of GHS with 10 points was found in 241 patients (53.4%). However, in the HCC and ICC cohorts, GHS was largely maintained during the first 2 follow-up periods (Fig. 1). The mCRC cohort showed similar results at the first follow-up period, but GHS was significantly reduced at the second follow-up period (*p* = 0.002). Supplement 5 shows a strong correlation between the deterioration of QOL over different dimensions, including GHS, and an increase in overall AEBS (*p* values ranging from < 0.001 to 0.0199). Univariable analysis (Table 6) shows that ECOG 0, > 1 TARE treatment and partition model were associated with ≥ 10 points deterioration of QOL (*p* = 0.0027, *p* = 0.0014 and *p* = 0.0041, respectively).

**Table 6:** Predictors for a deterioration of quality of life

Variable	Type	< 10 points deterioration	≥ 10 points deterioration	All	*P* value
Gender	Female	67 (34.0%)	81 (33.6%)	148 (33.8%)	0.4399
	Male	123 (62.4%)	156 (64.7%)	279 (63.7%)	
	Unknown	7 (3.6%)	4 (1.7%)	11 (2.5%)	
ECOG	0	122 (62.6%)	183 (76.3%)	305 (70.1%)	**0.0027**
	1	60 (30.8%)	52 (21.7%)	112 (25.7%)	
	≥ 2	13 (6.7%)	5 (2.1%)	18 (4.1%)	
Prior chemotherapy	No	113 (57.4%)	133 (55.2%)	246 (56.2%)	0.6483
	Yes	84 (42.6%)	108 (44.8%)	192 (43.8%)	
Prior locoregional treatments	No	118 (59.9%)	144 (59.8%)	262 (59.8%)	0.975
	Yes	79 (40.1%)	97 (40.2%)	176 (40.2%)	
Number of TARE treatments	1 TARE	168 (85.3%)	175 (72.6%)	343 (78.3%)	**0.0014**
	2 or more TARE	29 (14.7%)	66 (27.4%)	95 (21.7%)	
Location of liver tumours	Left	23 (25.0%)	29 (21.5%)	52 (22.9%)	0.5357
	Right	69 (75.0%)	106 (78.5%)	175 (77.1%)	
Prophylactic embolization	No	96 (65.3%)	140 (70.7%)	236 (68.4%)	0.2859
	Yes	51 (34.7%)	58 (29.3%)	109 (31.6%)	
Dose methodology	Partition Model	75 (38.7%)	125 (52.5%)	200 (46.3%)	**0.0041**
	BSA/mBSA	119 (61.3%)	113 (47.5%)	232 (53.7%)	

Analyses were performed using the Wilcoxon rank-sum test if the number of groups is 2 or with the Kruskal–Wallis test (non-parametric alternative to the ANOVA) if the number of groups exceed 2

*BSA* body surface area, *ECOG* Eastern Cooperative Oncology Group, *mBSA* modified body surface area, *TARE* transarterial radioembolization

## Discussion

This large prospective multicentre study describes how TARE treatments with Y90 resin microspheres are performed in patients with primary and metastatic liver disease and shows that regardless of indication, experienced interventional radiologists can apply the treatment safely with a low occurrence of severe AEs and low AEBS. We identified several predictors for an increased AEBS regardless of cancer type, described the predictors of an increased AEBS for HCC, ICC and colorectal liver metastases, specifically, and determined predictors for a deterioration of GHS. We furthermore established that a strong correlation between the AEBS and GHS exists.

### Application-Related Adverse Events

Severe AEs associated with the technical application of TARE are gastritis, gastrointestinal ulcerations, radiation cholecystitis, radiation pancreatitis, radiation pneumonitis and REILD [20]. In our cohort, we found that each of these non-target radiation AEs occurred in less than 2% of the patients. We reported that 13/1027 patients (1.3%) experienced REILD, of which half were grade 3 or higher. This occurrence of REILD is on the lower end of the range reported in a systematic review, which identified that the incidence of symptomatic REILD varied between 0 and 31%, although, in most reports, the incidence was 0–8% [25]. Furthermore, our cohort reported no occurrences of radiation pneumonitis, which is consistent with the generally low median lung shunt findings of 5–7% [26]. Supplement 6 discusses the severe AEs in more detail.

### Prognostic Factors for Adverse Events

Prior studies that identified prognostic factors for AEs were based on small retrospective cohorts [27–29]. The exception was the RESIN study, which included 614 patients with various indications and reported on predictors for AEs within 6 months after treatment [30].

In HCC, we found that ECOG 0, number of prior systemic treatments, 1 tumour nodule, number of TARE treatments, partition model dosimetry, and unilobar treatment target were associated with an increase in AEBS. Prior studies evaluating prognostic factors for safety and toxicity reported on severe AEs and commonly ignored the burden of symptomatic post-embolization AEs [27–29]. While the randomised trials SIRveNIB and SARAH reported adverse events of all grades [31, 32], comparing the complete safety profiles of TARE and sorafenib is complicated without the use of a numeric metric such as the AEBS. Furthermore, it should be noted that important differences in safety outcomes after TARE with glass or resin in HCC have been described [33].

In mCRC, our findings that > 2 lines of prior chemotherapy before TARE increased the AEBS reflect the current understanding regarding hepatotoxicities following oxaliplatin and irinotecan [34, 35]. Also here, we found ECOG 0 and 1 tumour nodule to be predictive of an increased AEBS, whereby the impact of low tumour volume on toxicity outcomes has been described in the RESIN study as well [30].

Interestingly, our cohort-wide findings suggest that partition model dosimetry predicts an increase in AEBS. This finding is not completely understood. Our data show that patients treated with partition model dosimetry had fewer cases of gastritis, gastrointestinal ulcerations, REILD, radiation pancreatitis or radiation cholecystitis, but presented more symptomatic adverse events than patients treated with body surface area (BSA) activity calculation methods. While partition model has demonstrated to increase the tumour-absorbed dose compared to standard activity calculations [36], several studies showed that an increase in tumour-absorbed dose was not associated with an increase in toxicity and that instead the level of toxicity was associated with an increase in parenchyma-absorbed dose [27, 37, 38]. Furthermore, it has been demonstrated that partition model dosimetry is associated with an increased survival [39], which means that patients may receive further treatments that cause additional AEs.

We also reported that patients with ECOG 0, unilobar treatments and 1 tumour nodule have an increased AEBS. As the data in Supplement 4 shows, these healthier patients reported more symptomatic AEs within the first month after treatment. We can hypothesise that patients with low tumour burden and fewer cancer-related symptoms are more aware of changes in their well-being and thus more likely to report mild symptomatic adverse events compared to patients with increased tumour-related symptoms or experience with prior treatments. Moreover, especially in our HCC cohort, 55.2% of the patients did not receive prior hepatic procedures and 89.3% did not receive prior systemic therapy [22], meaning that a substantial number of HCC patients had little experience with cancer treatments. Our results show that these healthier patients do report an increased number of post-TARE symptomatic adverse events. Indeed, some studies have shown that psychological distress and poor communication can lead to an increase in patient-reported symptomatic AEs [40, 41].

### Quality of Life

This prospective study shows that TARE has a minimum effect on QOL. Predictors for a deterioration of GHS were ECOG 0, > 1 TARE treatment and partition model dosimetry. Previous studies have shown that patients treated with TARE for HCC maintain a stable quality-of-life outcome [10, 42, 43] and TARE is considered favourable to TACE in that respect [44–46]. This is consistent with our findings, which shows no deterioration of GHS for HCC and ICC, and a minor deterioration of GHS after 6 months in the mCRC cohort. An extended discussion of quality of life after TARE can be found in Supplement 6.

### Limitations

The patient population included in this study is heterogeneous in presentation, treatment history and treatment pathway following TARE. Therefore, it should be considered that reported AEs might have been influenced by other treatments, or, vice versa, treatment-related AEs were considered unrelated due to concomitant therapies. In general, AEs are known to be underreported, both in clinical studies and to regulatory bodies [47–49]. Moreover, interventional radiology departments did not always have the appropriate infrastructure to consistently perform follow-ups, which contributed to the increased number of censored patients during follow-up and may have contributed to a potential underreporting of AEs and decreased returns of QOL questionnaires. Furthermore, the commonality of post-embolization syndrome may have caused some investigators to consider it as an expected side effect of TARE, instead of a reportable AE. Finally, the TARE treatments were performed between 2015 and 2017. Changes in practice and that investigator-determined follow-up regimes were not always optimal for detecting early AEs may not be reflected in the data. Despite the large number of patients without any reported AEs and potential missing data, our sample displays a high degree of internal validity as suggested by the strong correlation between an increase of overall AEBS and deterioration of QOL.

When interpreting the predictors for AEBS outcomes, it should be considered that for this analysis we combined all reported AEs in the follow-up period. Despite our findings that AEs in the “other” category were primarily occurring > 4 months after treatment, the exclusion of other AEs from the AEBS analysis could introduce a bias. We, therefore, supplemented this analysis by presenting adverse events outcomes per time interval (< 1 month, 1–4 months, and > 4 months after TARE). However, potential discrepancies in the number of follow-ups per time interval when comparing variables were not evaluated.

Finally, data points that may affect occurrences of REILD or other serious AEs, such as liver reserve, treated liver volume, or liver absorbed dose were not collected during the study, and their confounding impact should be considered when interpreting the results.

## Conclusion

This large prospective dataset on the use of TARE with Y90 resin microspheres shows that, in the real-world setting, TARE is very safe with minimal impact on GHS. It was established that the AEBS is a viable way to report on AEs and find prognostic factors for safety-related outcomes and correlates with deterioration of GHS. Predictors for an increased AEBS are cancer type, > 2 prior systemic treatments, multiple TARE treatments, no prophylactic embolization, ECOG 0, low tumour burden and partition model dosimetry. Predictors for a deterioration of GHS were ECOG 0, > 1 TARE treatment and partition model dosimetry. Further studies on intra-arterial treatments are encouraged to use the AEBS to accurately evaluate predictors for safety outcomes and improve patient safety.

## Supplementary Information

Below is the link to the electronic supplementary material.

Supplementary file1 (DOCX 102 KB)

## Abbreviations

99mTC MAA: Technetium 99m macroaggregated albumin
AE: Adverse event
AEBS: Adverse event burden score
ALT: Alanine transaminase
AST: Aspartate aminotransferase
BSA: Body surface area
CIRSE: Cardiovascular and Interventional Radiological Society of Europe
CIRT: CIRSE registry for SIR-spheres therapy
CTCAE: Common terminology criteria for AEs
ECOG: Eastern Cooperative Oncology Group
EORTC: European Organisation for the Research and Treatment of Cancer
GBq: Giga-becquerel
GHS: Global Health Score
HAI: Hepatic arterial infusion
HCC: Hepatocellular carcinoma
ICC: Intrahepatic cholangiocarcinoma
INR: International normalised ratio
IQR: Interquartile range
mBSA: Modified body surface area
mCRC: Metastatic colorectal cancer
QOL: Quality of life
RCT: Randomised controlled trial
REILD: Radioembolization-induced liver disease
SD: Standard deviation
SIRT: Selective internal radiation therapy
TACE: Transarterial chemoembolization
TARE: Transarterial radioembolization
Y90: Yttrium-90
