# Supplementary material for: Predictive Factors for Adverse Event Outcomes After Transarterial Radioembolization with Yttrium-90 Resin Microspheres in Europe: Results from the Prospective Observational CIRT Study
Source: Cardiovasc Intervent Radiol. 2023 Mar 13;46(7):852–67. doi: 10.1007/s00270-023-03391-4 (PMC10322946; doi:10.1007/s00270-023-03391-4)
Supplement: Supplementary file 1 — Supplementary file1 (DOCX 102 KB) [file 270_2023_3391_MOESM1_ESM.docx]

Supplementary information to

**Predictive factors for adverse events outcomes after transarterial radioembolization with Y-90 resin microspheres in Europe – results from the prospective observational CIRT study**

Contents

[Supplement 1: Adverse events burden score 2](#_Toc123738962)

[Supplement 2: Adverse events per time interval for intrahepatic cholangiocarcinoma, colorectal liver metastases and hepatocellular carcinoma 4](#_Toc123738963)

[Supplement 3: Univariable analysis of predictors of increased overall EABS in the whole cohort (A) in hepatocellular carcinoma (B), intrahepatic cholangiocarcinoma (C) and metastatic colorectal cancer (D) 6](#_Toc123738964)

[Supplement 4: Comparing occurrences of adverse events per time interval for ECOG, number of tumour nodules, tumour location, dose methodologies and prophylactic embolization 11](#_Toc123738965)

[Supplement 5: Association between AEBS and QOL deterioration 14](#_Toc123738966)

[Supplement 6: Extended discussion 15](#_Toc123738967)

# **Supplement 1: Adverse events burden score**

To compare the severity of treatment-related adverse events with non-treatment-related adverse events experienced by patients receiving TARE, and to identify prognostic factors for adverse events, we analysed the adverse events in a numeric fashion according to the overall adverse events burden score (OAEBS) proposed by La-Rademacher (2020). The AE burden by treatment cycle is a weighted sum of all grades and AEs that the patient experienced in a cycle. The overall AE burden score is the total AE burden the patient experienced across all treatment cycles.

Let $Y_{kg}(t)$ be the indicator that a patient experiences an AE of type *k* (= 1, … , *K*), grade *g* (= 1, … , 5), where *g* = 5 indicates death due to AE type *k*, at time (or treatment cycle) *t*. That is,

$$Y_{kg}(t)=\left\{ \begin{matrix} 1, if the patient experiences event k of grade g at time t \\ 0, \mathrm{otherwise} \end{matrix} \right.$$

We define the patient AE burden at time *t* as

$$B(t)=\sum_{k} \sum_{g} w_{kg}Y_{kg}\left( t \right)$$

a weighted sum of $Y_{kg}(t)$ values over all grades and AEs of interest. The definition requires a pre-specified severity weight, $w_{kg}$, for each combination of adverse event k and grade g. The values of ($w_{kg}$, k = 1, … , K, g = 1, … , 5) are subjective, and they serve the dual purposes of quantifying how bad one considers a combination (*k*, *g*) to be, and putting the severities of qualitatively different toxicities on the same numerical domain.

An overall AE burden score across treatment cycles/times is defined by summing over *t* as:

$$TB=\sum_{t} \mu_{t}[\sum_{k} \sum_{g} w_{kg}Y_{kg}\left( t \right)].$$

The weight function for $w_{kg}$is $w_{kg}=$ g, i.e., the weight of an AE equals the grade of the event regardless of *k*. The weight for grade 5 events will be inflated relative to the weights of grade 1-4 events as deemed appropriate for a specific tumour type and/or trial setting. A weight of 10 to grade 5 events will then be considered. This choice of weight for grade 5 events was intended to reflect the increased burden of death due to the AE (counts as twice its severity), while at the same time not being so large that it overshadows the burden of lower grade events.

A *TB* with the weight function $\mu_{t}=$ 1/c, where c is the number of treatments the patient received, equates to the overall AE burden a patient experienced averaged across all treatments.

For the calculation of the overall AEBS, adverse events that were ungraded were excluded. Adverse events that were included in the “Other” category were also excluded from the analysis on the grounds of lacking consistent gradings. Finally, abnormal laboratory values were excluded from the AEBS analysis because they were not considered to be causing patient suffering (i.e., a grade 1 hyperbilirubinemia would not be noticeable to a patient while a grade 1 abdominal pain would be).

# **Supplement 2: Adverse events per time interval for intrahepatic cholangiocarcinoma, colorectal liver metastases and hepatocellular carcinoma**

| **At least 1 AE per time interval**  n (%) | | | |
| --- | --- | --- | --- |
|  | <1 month | 1-4 months | >4 months |
| **Cholangiocarcinoma** | | | |
| N all AEs | 57 | 52 | 122 |
| Number of treatments | 144 | 144 | 144 |
| Number of patients with a follow-up in this interval | 108 | 89 | 76 |
| Abdominal pain | 16 (14.8) | 11 (12.4) | 17 (22.4) |
| Fatigue | 15 (13.9) | 11 (12.4) | 20 (26.3) |
| Fever | NA | 3 (3.4) | 2 (2.6) |
| Nausea | 8 (7.4) | 6 (6.7) | 10 (13.2) |
| Vomiting | 7 (6.5) | 1 (1.1) | 4 (5.3) |
| Gastrointestinal ulceration | 1 (0.9) | 4 (4.5) | 1 (1.3) |
| Gastritis | 1 (0.9) | 1 (1.1) | NA |
| Radiation cholecystitis | NA | 1 (1.1) | NA |
| Radioembolization-induced liver disease | NA | 3 (3.4) | 1 (1.3) |
| Other | NA | 4 (4.5) | 21 (27.6) |
| **Colorectal Cancer** | | | |
| N all AEs | 108 | 86 | 164 |
| Number of treatments | 264 | 264 | 264 |
| Number of patients with a follow-up in this interval | 195 | 151 | 123 |
| Abdominal pain | 29 (14.9) | 14 (9.3) | 19 (15.4) |
| Fatigue | 17 (8.7) | 16 (10.6) | 23 (18.7) |
| Fever | 4 (2.1) | 5 (3.3) | 2 (1.6) |
| Nausea | 13 (6.7) | 11 (7.3) | 12 (9.8) |
| Vomiting | 9 (4.6) | 3 (2) | 3 (2.4) |
| Gastrointestinal ulceration | 1 (0.5) | NA | 2 (1.6) |
| Gastritis | 4 (2.1) | 2 (1.3) | 1 (0.8) |
| Radiation cholecystitis | 1 (0.5) | NA | NA |
| Radioembolization-induced liver disease | NA | 1 (0.7) | NA |
| Other | 7 (3.6) | 19 (12.6) | 38 (30.9) |
| **Hepatocellular Carcinoma** | | | |
| N all AEs | 144 | 90 | 301 |
| Number of treatments | 496 | 496 | 496 |
| Number of patients with a follow-up in this interval | 387 | 318 | 292 |
| Abdominal pain | 33 (8.5) | 16 (5) | 35 (12) |
| Fatigue | 39 (10.1) | 20 (6.3) | 53 (18.2) |
| Fever | 6 (1.6) | 7 (2.2) | 11 (3.8) |
| Nausea | 20 (5.2) | 11 (3.5) | 15 (5.1) |
| Vomiting | 15 (3.8) | 4 (1.3) | 7 (2.4) |
| Gastrointestinal ulceration | NA | 2 (0.6) | 1 (0.3) |
| Gastritis | 1 (0.3) | 1 (0.3) | NA |
| Radiation cholecystitis | NA | NA | NA |
| Radioembolization-induced liver disease | 1 (0.3) | 4 (1.3) | NA |
| Other | 8 (2.1) | 17 (5.3) | 59 (20.2) |

# **Supplement 3: Univariable analysis of predictors of increased overall EABS in the whole cohort (A) in hepatocellular carcinoma (B), intrahepatic cholangiocarcinoma (C) and metastatic colorectal cancer (D)**

**A: Univariable analysis of predictors of increased overall AEBS for the whole cohort**

| **Variable** | **Categories** | **N** | **Mean AEBS ± SD** | **P-value** |
| --- | --- | --- | --- | --- |
| Gender | Male | 352 | 1.6 ± 3.4 | 0.183 |
|  | Female | 655 | 1.3 ± 3.4 |  |
|  | Unknown | 20 | 0.7 ± 1.6 |  |
| Cancer type | Hepatocellular carcinoma | 422 | 1.2 ± 3.8 | **0.0422** |
|  | Intrahepatic cholangiocarcinoma | 120 | 2.0 ± 4.7 |  |
|  | Colorectal cancer liver mets | 237 | 1.2 ± 3.1 |  |
|  | Neuroendocrine tumour liver mets | 58 | 2.3 ± 5.5 |  |
|  | Breast cancer liver mets | 47 | 2.2 ± 4.7 |  |
|  | Pancreatic cancer liver mets | 32 | 1.1 ± 1.5 |  |
|  | Melanoma liver mets | 32 | 1.2 ± 1.8 |  |
| **ECOG** | **0** | **625** | **1.9 ± 4.5** | **<0.0001** |
|  | 1 | 316 | 0.7 ± 1.8 |  |
|  | ≥2 | 75 | 0.4 ± 1.4 |  |
| Extra-hepatic disease | No | 732 | 1.5 ± 4.0 | 0.9071 |
|  | Yes | 295 | 1.2 ± 3.0 |  |
| Prior systemic therapy | No | 516 | 1.3 ± 3.9 | 0.1644 |
|  | Yes | 511 | 1.5 ± 3.6 |  |
| **Number of prior treatments** | 0 | 521 | 1.3 ± 3.9 | **<0.0001** |
|  | 1 | 191 | 0.5 ± 1.4 |  |
|  | 2 | 98 | 0.8 ± 2.0 |  |
|  | **>2** | **217** | **2.6 ± 5.0** |  |
| Prior hepatic procedures | No | 633 | 1.4 ± 3.7 | 0.3936 |
|  | Yes | 393 | 1.3 ± 3.8 |  |
| Location of liver tumours | Bilobar | 561 | 1.1 ± 2.9 | 0.2328 |
|  | Left | 101 | 1.9 ± 4.3 |  |
|  | Right | 364 | 1.7 ± 4.6 |  |
| **Number of tumour nodules** | **1** | **230** | **2.5 ± 5.4** | **0.0002** |
|  | 2-5 | 253 | 1.2 ± 3.4 |  |
|  | >5 | 245 | 1.3 ± 3.3 |  |
|  | Uncountable | 299 | 0.8 ± 2.5 |  |
| Percentage tumour invasion in the liver (whole liver) | <10% | 276 | 0.9 ± 3.2 | 0.0589 |
|  | 10-20% | 139 | 0.8 ± 1.8 |  |
|  | >20% | 156 | 0.6 ± 1.6 |  |
| Percentage tumour invasion in the liver (right lobe) | <10% | 196 | 2.2 ± 4.6 | 0.752 |
|  | 10-20% | 99 | 2.7 ± 6.1 |  |
|  | >20% | 103 | 2.2 ± 4.4 |  |
| **Percentage tumour invasion in the liver (left lobe)** | <10% | 224 | 2.1 ± 4.9 | **0.0283** |
|  | 10-20% | 73 | 2.2 ± 4.9 |  |
|  | **>20%** | **85** | **3.5 ± 5.5** |  |
| **Number of TARE treatments** | 1 | 869 | 1.2 ± 3.4 | **<.0001** |
|  | **≥2** | **158** | **2.6 ± 5.2** |  |
| Unilobar treatment | Left | 101 | 1.9 ± 3.4 | 0.4652 |
|  | Right | 364 | 1.7 ± 4.5 |  |
| **Prophylactic embolization** | Yes | 412 | 0.8 ± 2.1 | **0.0222** |
|  | **No** | **477** | **2.0 ± 4.9** |  |
| **Dose methodology** | BSA/mBSA | 736 | 1.1 ± 3.2 | **0.0022** |
|  | **Partition** | **279** | **2.1 ± 4.9** |  |
| **TARE Target** | **Left lobe** | **124** | **2.2 ± 4.6** | **0.0448** |
|  | **Right lobe** | **337** | **1.7 ± 4.2** |  |
|  | Whole liver (segmental) | 98 | 0.9 ± 3.3 |  |
|  | Whole liver (sequential) | 136 | 1.0 ± 2.4 |  |
|  | Whole liver (single catheter) | 121 | 1.1 ± 3.7 |  |
|  | Whole liver (split administration) | 171 | 1.1 ± 2.9 |  |
| Prescribed activity (GBq) | <1 | 252 | 1.4 ± 3.5 | 0.133 |
|  | 1.1-1.5 | 260 | 1.8 ± 4.9 |  |
|  | 1.5-1.82 | 257 | 0.9 ± 2.5 |  |
|  | >1.82 | 257 | 1.5 ± 3.8 |  |
| Analyses were performed using the Wilcoxon Rank Sum test if the number of groups is 2, or with the Kruskal–Wallis test (non-parametric alternative to the ANOVA) if the number of groups exceed 2. Multivariable analysis could not be performed due to the high number of patients with no adverse events.  BSA: body surface area; ECOG: Eastern Cooperative Oncology Group; GBq: giga-becquerel; mBSA: modified body surface area; SD: standard deviation; TARE: transarterial radioembolization. | | | | |

**B: Univariable analysis of predictors of increased overall AEBS in HCC**

| **Variable** | **Categories** | **N** | **Mean AEBS ± SD** | **P-value** |
| --- | --- | --- | --- | --- |
| Gender | Male | 341 | 1.3 ± 4.9 | 0.3785 |
|  | Female | 74 | 1.2 ± 3.6 |  |
|  | Unknown | 7 | 0.0 ± 0.0 |  |
| ECOG | 0 | 260 | 1.7 ± 4.7 | 0.0045 |
|  | 1 | 131 | 0.6 ± 1.5 |  |
|  | ≥2 | 31 | 0.0 ± 0.1 |  |
| Extra-hepatic disease | No | 386 | 1.2 ± 3.9 | 0.5552 |
|  | Yes | 36 | 1.3 ± 2.9 |  |
| Cirrhosis | No | 123 | 0.8 ± 3.4 | 0.1939 |
|  | Yes | 299 | 1.4 ± 4.0 |  |
| Prior systemic therapy | No | 381 | 1.3 ± 3.9 | 0.712 |
|  | Yes | 41 | 1.0 ± 2.7 |  |
| Number of prior treatments | 0 | 381 | 1.3 ± 3.9 | 0.0107 |
|  | 1 | 35 | 0.6 ± 2.2 |  |
|  | 2 | 4 | 1.0 ± 2.0 |  |
|  | >2 | 2 | 8.5 ± 0.7 |  |
| Prior hepatic procedures | No | 233 | 1.2 ± 4.2 | 0.5283 |
|  | Yes | 189 | 1.3 ± 3.8 |  |
| Location of liver tumours | Bilobar | 150 | 0.7 ± 1.9 | 0.3955 |
|  | Left | 51 | 1.9 ± 4.8 |  |
|  | Right | 221 | 1.5 ± 4.5 |  |
| Number of tumour nodules | 1 | 136 | 2.2 ± 5.0 | 0.0081 |
|  | 2-5 | 138 | 1.0 ± 3.5 |  |
|  | >5 | 72 | 1.0 ± 3.4 |  |
|  | Uncountable | 76 | 0.3 ± 1.1 |  |
| Percentage tumour invasion in the liver (whole liver) | <10% | 105 | 0.7 ± 3.6 | 0.5504 |
|  | 10-20% | 49 | 0.6 ± 1.5 |  |
|  | >20% | 63 | 0.9 ± 2.1 |  |
| Percentage tumour invasion in the liver (right lobe) | <10% | 102 | 1.7 ± 4.0 | 0.4294 |
|  | 10-20% | 32 | 2.3 ± 6.8 |  |
|  | >20% | 39 | 2.2 ± 4.7 |  |
| Percentage tumour invasion in the liver (left lobe) | <10% | 118 | 1.8 ± 4.8 | 0.1284 |
|  | 10-20% | 19 | 0.9 ± 1.8 |  |
|  | >20% | 32 | 3.2 ± 5.8 |  |
| Number of TARE treatments | 1 | 353 | 1.2 ± 3.8 | 0.0224 |
|  | ≥2 | 69 | 1.6 ± 3.9 |  |
| Unilobar treatment | Left | 51 | 1.9 ± 4.8 | 0.4024 |
|  | Right | 221 | 1.5 ± 4.5 |  |
| Prophylactic embolization | No | 217 | 1.8 ± 4.9 | 0.0211 |
|  | Yes | 138 | 0.7 ± 2.2 |  |
| Dose methodology | BSA/mBSA | 245 | 0.7 ± 2.8 | 0.0007 |
|  | Partition | 173 | 2.0 ± 4.8 |  |
| TARE Target | Left lobe | 56 | 1.8 ± 4.6 | 0.0032 |
|  | Right lobe | 177 | 1.6 ± 4.2 |  |
|  | Whole liver (segmental) | 66 | 0.2 ± 0.9 |  |
|  | Whole liver (sequential) | 33 | 1.1 ± 2.8 |  |
|  | Whole liver (single catheter) | 53 | 1.4 ± 5.1 |  |
|  | Whole liver (split administration) | 37 | 0.2 ± 0.9 |  |
| Prescribed activity (GBq) | <1 | 104 | 1.5 ± 3.8 | 0.105 |
|  | 1.1-1.5 | 99 | 1.0 ± 3.3 |  |
|  | 1.5-1.82 | 111 | 0.9 ± 3.8 |  |
|  | >1.82 | 108 | 1.5 ± 4.3 |  |
| Analyses were performed using the Wilcoxon Rank Sum test if the number of groups is 2, or with the Kruskal–Wallis test (non-parametric alternative to the ANOVA) if the number of groups exceed 2. Multivariable analysis could not be performed due to the high number of patients with no adverse events.  BSA: body surface area; ECOG: Eastern Cooperative Oncology Group; GBq: giga-becquerel; mBSA: modified body surface area; SD: standard deviation; TARE: transarterial radioembolization. | | | | |

**C: Univariable analysis of predictors of increased overall AEBS in ICC**

| **Variable** | **Categories** | **N** | **Mean AEBS ± SD** | **P-value** |
| --- | --- | --- | --- | --- |
| Gender | Male | 65 | 1.5 ± 3.4 | 0.2207 |
|  | Female | 51 | 2.7 ± 6.0 |  |
|  | Unknown | 4 | 0.0 ± 0.0 |  |
| ECOG | 0 | 69 | 2.8 ± 5.8 | 0.143 |
|  | 1 | 39 | 0.6 ± 1.5 |  |
|  | ≥2 | 9 | 1.8 ± 3.2 |  |
| Extra-hepatic disease | No | 85 | 2.4 ± 5.3 | 0.3336 |
|  | Yes | 35 | 1.0 ± 2.1 |  |
| Prior systemic therapy | No | 51 | 2.0 ± 5.2 | 0.5933 |
|  | Yes | 69 | 1.9 ± 4.3 |  |
| Number of prior treatments | 0 | 52 | 2.1 ± 5.2 | 0.0996 |
|  | 1 | 34 | 0.6 ± 1.2 |  |
|  | 2 | 15 | 0.6 ± 1.2 |  |
|  | >2 | 19 | 5.2 ± 7.0 |  |
| Prior hepatic procedures | No | 79 | 2.2 ± 5.3 | 0.3975 |
|  | Yes | 41 | 1.5 ± 3.2 |  |
| Location of liver tumours | Bilobar | 68 | 1.8 ± 4.2 | 0.8949 |
|  | Left | 17 | 1.6 ± 3.3 |  |
|  | Right | 35 | 2.4 ± 5.9 |  |
| Number of tumour nodules | 1 | 44 | 3.3 ± 6.5 | 0.0773 |
|  | 2-5 | 25 | 1.6 ± 4.2 |  |
|  | >5 | 20 | 1.3 ± 2.2 |  |
|  | Uncountable | 31 | 1.0 ± 2.2 |  |
| Percentage tumour invasion in the liver (whole liver) | <10% | 27 | 1.3 ± 3.4 | 0.1346 |
|  | 10-20% | 27 | 1.0 ± 2.1 |  |
|  | >20% | 23 | 0.2 ± 0.7 |  |
| Percentage tumour invasion in the liver (right lobe) | <10% | 15 | 5.4 ± 7.4 | 0.7782 |
|  | 10-20% | 10 | 4.9 ± 9.2 |  |
|  | >20% | 10 | 2.7 ± 4.9 |  |
| Percentage tumour invasion in the liver (left lobe) | <10% | 16 | 3.6 ± 7.6 | 0.8789 |
|  | 10-20% | 9 | 5.6 ± 8.5 |  |
|  | >20% | 12 | 4.3 ± 5.7 |  |
| Number of TARE treatments | 1 | 97 | 1.6 ± 3.5 | 0.0224 |
|  | ≥2 | 23 | 3.7 ± 7.8 |  |
| Unilobar treatment | Left | 17 | 1.6 ± 3.3 | 0.7979 |
|  | Right | 35 | 2.4 ± 5.9 |  |
| Prophylactic embolization | No | 61 | 2.5 ± 5.9 | 0.6011 |
|  | Yes | 39 | 1.0 ± 1.8 |  |
| Dose methodology | BSA/mBSA | 79 | 1.8 ± 4.2 | 0.3026 |
|  | Partition | 39 | 2.4 ± 5.7 |  |
| TARE Target | Left lobe | 20 | 3.9 ± 6.3 | 0.442 |
|  | Right lobe | 34 | 2.3 ± 5.9 |  |
|  | Whole liver (segmental) | 11 | 0.2 ± 0.6 |  |
|  | Whole liver (sequential) | 17 | 1.2 ± 3.1 |  |
|  | Whole liver (single catheter) | 13 | 1.2 ± 2.5 |  |
|  | Whole liver (split administration) | 25 | 1.7 ± 3.6 |  |
| Prescribed activity (GBq) | <1 | 28 | 1.4 ± 2.8 | 0.5602 |
|  | 1.1-1.5 | 32 | 3.5 ± 7.3 |  |
|  | 1.5-1.82 | 30 | 1.5 ± 3.5 |  |
|  | >1.82 | 30 | 1.3 ± 3.2 |  |
| Analyses were performed using the Wilcoxon Rank Sum test if the number of groups is 2, or with the Kruskal–Wallis test (non-parametric alternative to the ANOVA) if the number of groups exceed 2. Multivariable analysis could not be performed due to the high number of patients with no adverse events.  BSA: body surface area; ECOG: Eastern Cooperative Oncology Group; GBq: giga-becquerel; mBSA: modified body surface area; SD: standard deviation; TARE: transarterial radioembolization. | | | | |

**D: Univariable analysis of predictors of increased overall AEBS in mCRC**

| **Variable** | **Categories** | **N** | **Mean AEBS ± SD** | **P-value** |
| --- | --- | --- | --- | --- |
| Gender | Male | 147 | 1.2 ± 3.2 | 0.3762 |
|  | Female | 86 | 1.2 ± 3.1 |  |
|  | Unknown | 4 | 2.5 ± 2.9 |  |
| ECOG | 0 | 143 | 1.8 ± 3.8 | 0.0188 |
|  | 1 | 72 | 0.5 ± 1.5 |  |
|  | ≥2 | 18 | 0.5 ± 1.1 |  |
| Extra-hepatic disease | No | 140 | 1.2 ± 3.1 | 0.7552 |
|  | Yes | 97 | 1.3 ± 3.2 |  |
| Prior systemic therapy | No | 18 | 0.8 ± 1.8 | 0.8648 |
|  | Yes | 219 | 1.3 ± 3.2 |  |
| Number of prior treatments | 0 | 21 | 1.1 ± 2.6 | 0.0127 |
|  | 1 | 69 | 0.3 ± 1.0 |  |
|  | 2 | 41 | 0.7 ± 2.1 |  |
|  | >2 | 106 | 2.1 ± 4.1 |  |
| Prior hepatic procedures | No | 150 | 1.2 ± 3.1 | 0.8025 |
|  | Yes | 86 | 1.4 ± 3.2 |  |
| Location of liver tumours | Bilobar | 152 | 0.9 ± 2.2 | 0.3521 |
|  | Left | 23 | 1.0 ± 1.9 |  |
|  | Right | 61 | 2.2 ± 4.9 |  |
| Number of tumour nodules | 1 | 27 | 2.5 ± 4.8 | 0.0155 |
|  | 2-5 | 50 | 1.7 ± 3.5 |  |
|  | >5 | 79 | 1.4 ± 3.2 |  |
|  | Uncountable | 81 | 0.4 ± 1.4 |  |
| Percentage tumour invasion in the liver (whole liver) | <10% | 72 | 1.0 ± 2.7 | 0.1763 |
|  | 10-20% | 35 | 1.2 ± 2.4 |  |
|  | >20% | 23 | 0.3 ± 1.1 |  |
| Percentage tumour invasion in the liver (right lobe) | <10% | 44 | 2.5 ± 4.7 | 0.2407 |
|  | 10-20% | 28 | 1.5 ± 3.8 |  |
|  | >20% | 26 | 0.7 ± 2.2 |  |
| Percentage tumour invasion in the liver (left lobe) | <10% | 51 | 2.4 ± 5.0 | 0.441 |
|  | 10-20% | 25 | 1.1 ± 2.6 |  |
|  | >20% | 16 | 0.9 ± 1.7 |  |
| Number of TARE treatments | 1 | 210 | 1.1 ± 2.8 | 0.1066 |
|  | ≥2 | 27 | 2.5 ± 4.7 |  |
| Unilobar treatment | Left | 23 | 1.0 ± 1.9 | 0.7121 |
|  | Right | 61 | 2.2 ± 4.9 |  |
| Prophylactic embolization | No | 95 | 1.6 ± 4.1 | 0.9899 |
|  | Yes | 115 | 0.8 ± 2.0 |  |
| Dose methodology | BSA/mBSA | 194 | 1.2 ± 2.7 | 0.4152 |
|  | Partition | 37 | 1.9 ± 5.0 |  |
| TARE Target | Left lobe | 26 | 0.7 ± 1.7 | 0.8761 |
|  | Right lobe | 90 | 1.7 ± 4.2 |  |
|  | Whole liver (segmental) | 7 | 1.7 ± 3.1 |  |
|  | Whole liver (sequential) | 32 | 0.8 ± 1.7 |  |
|  | Whole liver (single catheter) | 28 | 0.8 ± 2.2 |  |
|  | Whole liver (split administration) | 54 | 1.2 ± 2.6 |  |
| Prescribed activity (GBq) | <1 | 55 | 1.8 ± 4.4 | 0.4437 |
|  | 1.1-1.5 | 63 | 1.1 ± 2.4 |  |
|  | 1.5-1.82 | 59 | 0.6 ± 1.8 |  |
|  | >1.82 | 59 | 1.5 ± 3.3 |  |
| Analyses were performed using the Wilcoxon Rank Sum test if the number of groups is 2, or with the Kruskal–Wallis test (non-parametric alternative to the ANOVA) if the number of groups exceed 2. Multivariable analysis could not be performed due to the high number of patients with no adverse events.  BSA: body surface area; ECOG: Eastern Cooperative Oncology Group; GBq: giga-becquerel; mBSA: modified body surface area; SD: standard deviation; TARE: transarterial radioembolization. | | | | |

# **Supplement 4: Comparing occurrences of adverse events per time interval for ECOG, number of tumour nodules, tumour location, dose methodologies and prophylactic embolization**

**A. ECOG**

| **ECOG** | | | | | | |
| --- | --- | --- | --- | --- | --- | --- |
| **Patients** | **ECOG 0 (625)** | | | **ECOG >0 (381)** | | |
| **Treatments*** | **ECOG 0 (631)** | | | **ECOG >0 (416)** | | |
| **Time interval** | **<1 month** | **1-4 months** | **>4 months** | **<1 month** | **1-4 months** | **>4 months** |
| **Patients with at least 1 follow-up per time-interval** | **565** | **463** | **421** | **366** | **271** | **222** |
| Abdominal pain | 103 (18.4) | 56 (12.1) | 77 (18.3) | 22 (6) | 9 (3.3) | 17 (7.7) |
| Fatigue | 94 (16.6) | 59 (12.7) | 110 (26.1) | 10 (2.8) | 14 (5.2) | 17 (7.7) |
| Fever | 8 (1.4) | 8 (1.7) | 14 (3.3) | 3 (0.8) | 10 (3.7) | 6 (2.7) |
| Nausea | 61 (10.8) | 29 (6.3) | 47 (11.2) | 7 (1.9) | 14 (5.2) | 2 (0.9) |
| Vomiting | 43 (7.6) | 10 (2.2) | 15 (3.6) | 5 (1.4) | 3 (1.1) | 1 (0.5) |
| Gastritis | 6 (1.1) | 3 (0.6) | 1 (0.2) | 1 (0.3) | 2 (0.7) | NA |
| Gastrointestinal ulceration | 2 (0.4) | 4 (0.9) | 3 (0.7) | 1 (0.3) | 2 (0.7) | 1 (0.5) |
| Radiation cholecystitis | 1 (0.2) | 2 (0.4) | 1 (0.2) | - | - | - |
| Radiation pancreatitis | - | 1 (0.2) | - | - | - | - |
| REILD | - | 2 (0.4) | 1 (0.2) | 2 (0.5) | 8 (3) | NA |
| Other | 13 (2.3) | 46 (9.9) | 114 (27.1) | 7 (1.9) | 16 (5.9) | 40 (18) |
| n (%), percentage is taken over the number of follow-ups during a time interval  * patients can have multiple treatments | | | | | | |

**B. Number of tumour nodules**

| **Number of tumour nodules** | | | | | | |
| --- | --- | --- | --- | --- | --- | --- |
| **Patients** | **1 nodule (230)** | | | **>1 (797)** | | |
| **Treatments*** | **1 nodule (241)** | | | **>1 (809)** | | |
| **Time interval** | **<1 month** | **1-4 months** | **>4 months** | **<1 month** | **1-4 months** | **>4 months** |
| **Patients with at least 1 follow-up per time-interval** | **226** | **185** | **166** | **706** | **551** | **479** |
| Abdominal pain | 43 (19) | 16 (8.6) | 40 (24.1) | 83 (11.8) | 49 (8.9) | 54 (11.3) |
| Fatigue | 43 (19) | 26 (14.1) | 61 (36.7) | 61 (8.6) | 49 (8.9) | 67 (14) |
| Fever | 3 (1.3) | 3 (1.6) | 7 (4.2) | 8 (1.1) | 16 (2.9) | 13 (2.7) |
| Nausea | 30 (13.3) | 10 (5.4) | 27 (16.3) | 39 (5.5) | 34 (6.2) | 23 (4.8) |
| Vomiting | 19 (7.9) | 4 (2.2) | 9 (5.4) | 30 (4.3) | 9 (1.6) | 7 (1.5) |
| Gastritis | 3 (1.3) | 1 (0.5) | NA | 5 (0.7) | 4 (0.7) | 1 (0.2) |
| Gastrointestinal ulceration | - | - | 1 (0.6) | 3 (0.4) | 6 (1.1) | 4 (0.8) |
| Radiation cholecystitis | - | - | - | 1 (0.1) | 1 (0.2) | - |
| Radiation pancreatitis | - | 1 (0.5) | - | - | 1 (0.2) | 1 (0.2) |
| REILD | 1 (0.4) | - | - | 1 (0.1) | 10 (1.8) | 1 (0.2) |
| Other | 3 (1.3) | 8 (4.3) | 41 (24.7) | 17 (2.4) | 54 (9.8) | 115 (24) |
| n (%), percentage is taken over the number of follow-ups during a time interval  * patients can have multiple treatments | | | | | | |

**C. Tumour location**

| **Tumour location** | | | | | | |
| --- | --- | --- | --- | --- | --- | --- |
| **Patients** | **Unilobar (465)** | | | **Bilobar (561)** | | |
| **Treatments*** | **Unilobar (492)** | | | **Bilobar (572)** | | |
| **Time interval** | **<1 month** | **1-4 months** | **>4 months** | **<1 month** | **1-4 months** | **>4 months** |
| **Patients with at least 1 follow-up per time-interval** | **453** | **370** | **342** | **493** | **372** | **308** |
| Abdominal pain | 73 (16.1) | 28 (7.6) | 62 (18.1) | 54 (10.7) | 37 (9.9) | 32 (10.4) |
| Fatigue | 73 (16.1) | 44 (11.9) | 89 (26) | 31 (6.3) | 31 (8.3) | 39 (12.7) |
| Fever | 8 (1.8) | 8 (2.2) | 13 (3.8) | 3 (0.6) | 11 (3) | 7 (2.3) |
| Nausea | 48 (10.6) | 19 (5.1) | 36 (10.5) | 21 (4.3) | 25 (6.7) | 14 (4.5) |
| Vomiting | 33 (7.3) | 5 (1.4) | 13 (3.8) | 15 (3) | 8 (2.2) | 3 (1) |
| Gastritis | 4 (0.9) | 1 (0.3) | 1 (0.3) | 4 (0.8) | 4 (1.1) | NA |
| Gastrointestinal ulceration | 1 (0.2) | - | - | 2 (0.4) | 6 (1.6) | 5 (1.6) |
| Radiation cholecystitis | 1 (0.2) | 1 (0.3) | - | - | 1 (0.3) | 1 (0.3) |
| Radiation pancreatitis | - | - | - | - | 1 (0.3) | - |
| REILD | 2 (0.4) | 2 (0.5) | - | NA | 8 (2.2) | 1 (0.3) |
| Other | 11 (2.4) | 18 (4.9) | 70 (20.5) | 9 (1.8) | 44 (11.8) | 86 (27.9) |
| n (%), percentage is taken over the number of follow-ups during a time interval  * patients can have multiple treatments | | | | | | |

**D. Dose methodology**

| **Dose methodology** | | | | | | |
| --- | --- | --- | --- | --- | --- | --- |
| **Patients** | **BSA (736)** | | | **Partition model (279)** | | |
| **Treatments*** | **BSA (738)** | | | **Partition model (289)** | | |
| **Time interval** | **<31 days** | **1-4 months** | **>4 months** | **<1 month** | **1-4 months** | **>4 months** |
| **Patients with at least 1 follow-up per time-interval** | **631** | **473** | **425** | **279** | **247** | **212** |
| Abdominal pain | 82 (12.7) | 46 (9.7) | 49 (11.5) | 45 (16.5) | 18 (7.3) | 45 (21.2) |
| Fatigue | 50 (7.9) | 42 (8.9) | 58 (13.6) | 54 (19.4) | 32 (13) | 70 (33) |
| Fever | 6 (1) | 13 (2.7) | 13 (3.1) | 5 (1.8) | 5 (2) | 7 (3.3) |
| Nausea | 38 (6) | 29 (6.1) | 20 (4.7) | 31 (11.1) | 15 (6.1) | 30 (14.2) |
| Vomiting | 28 (4.5) | 9 (1.9) | 6 (1.4) | 18 (6.5) | 4 (1.6) | 10 (4.7) |
| Gastritis | 7 (1.1) | 5 (1.1) | 1 (0.2) | 1 (0.4) | - | - |
| Gastrointestinal ulceration | 3 (0.5) | 6 (1.3) | 5 (1.2) | - | - | - |
| Radiation cholecystitis | 1 (0.2) | 2 (0.4) | 1 (0.2) | - | - | - |
| Radiation pancreatitis | - | 1 (0.2) | - | - | - | - |
| REILD | 1 (0.2) | 9 (1.9) | 1 (0.2) | 1 (0.4) | 1 (0.4) | - |
| Other | 18 (2.9) | 55 (11.6) | 104 (24.5) | 1 (0.4) | 5 (2) | 47 (22.2) |
| n (%), percentage is taken over the number of follow-ups during a time interval  * patients can have multiple treatments | | | | | | |

**E. Prophylactic embolization**

| **Prophylactic embolization** | | | | | | |
| --- | --- | --- | --- | --- | --- | --- |
| **Patients** | **Yes (412)** | | | **No (477)** | | |
| **Treatments*** | **Yes (419)** | | | **No (504)** | | |
| **Time interval** | **<1 month** | **1-4 months** | **>4 months** | **<1 month** | **1-4 months** | **>4 months** |
| **Patients with at least 1 follow-up per time-interval** | **346** | **251** | **236** | **461** | **367** | **328** |
| Abdominal pain | 33 (6.7) | 23 (9.2) | 15 (6.4) | 92 (20) | 38 (10.4) | 64 (19.5) |
| Fatigue | 7 (2) | 21 (8.4) | 20 (8.5) | 92 (20) | 51 (13.9) | 94 (28.7) |
| Fever | 3 (0.9) | 8 (3.2) | 7 (3) | 7 (1.5) | 9 (2.5) | 11 (3.4) |
| Nausea | 5 (1.5) | 20 (8) | 5 (2.1) | 60 (13) | 21 (5.7) | 43 (13.1) |
| Vomiting | 6 (1.8) | 6 (2.4) | 3 (1.3) | 40 (8.7) | 4 (1.1) | 12 (3.7) |
| Gastritis | 7 (2) | 4 (1.6) | 1 (0.4) | 1 (0.2) | - | - |
| Gastrointestinal ulceration | 3 (0.9) | 3 (1.2) | 4 (1.7) | - | - | - |
| Radiation cholecystitis | - | 2 (0.8) | - | 1 (0.2) | - | 1 (0.3) |
| Radiation pancreatitis | - | 1 (0.4) | - | - | - | - |
| REILD | - | 5 (2) | 1 (0.4) | 1 (0.2) | 1 (0.3) | - |
| Other | 8 (2.3) | 27 (10.8) | 60 (25.4) | 8 (1.7) | 17 (4.6) | 49 (14.9) |
| n (%), percentage is taken over the number of follow-ups during a time interval  * patients can have multiple treatments | | | | | | |

# **Supplement 5: Association between AEBS and QOL deterioration**

| **Variable** | **QOL deterioration** | **Patients (n)** | **AEBS (Mean ± SD)** | **P-value** |
| --- | --- | --- | --- | --- |
| Global health score | <10 points | 197 | 0.8 ± 1.8 | <0.0001 |
|  | **≥10 points** | **241** | **3.4 ± 6.0** |  |
| Physical functioning | <10 points | 184 | 0.7 ± 2.2 | <0.0001 |
|  | **≥10 points** | **267** | **3.2 ± 5.7** |  |
| Role functioning | <10 points | 172 | 0.8 ± 2.4 | <0.0001 |
|  | **≥10 points** | **270** | **3.2 ± 5.6** |  |
| Emotional functioning | <10 points | 230 | 1.4 ± 3.2 | 0.011 |
|  | **≥10 points** | **212** | **3.1 ± 6.0** |  |
| Cognitive functioning | <10 points | 269 | 1.7 ± 3.8 | 0.0199 |
|  | **≥10 points** | **171** | **3.0 ± 6.0** |  |
| Social functioning | <10 points | 174 | 1.2 ± 2.9 | 0.0007 |
|  | **≥10 points** | **266** | **2.9 ± 5.6** |  |
| AEBS: adverse events burden score; QOL: quality of life; SD: standard deviation | | | | |

# **Supplement 6: Extended discussion**

Application-related adverse events

Severe AEs associated with the technical application of TARE are gastritis, gastrointestinal ulcerations, radiation cholecystitis, radiation pancreatitis, radiation pneumonitis and REILD (1). In our cohort, we found that each of these non-target radiation AEs occurred in less than 2% of the patients, which indicates a good proficiency of the interventional radiologists applying the treatment. Indeed, it has been identified early on that prior evaluation of lung shunts using Technetium 99m macroaggregated albumin (^99m^Tc MAA) and careful assessment of the vessel locations using visceral angiography can identify non-hepatic arteries. Prophylactic coil or plug embolization of off-target vessels can prevent the unwanted distribution of the radioactive microspheres to the lung or gastrointestinal tract and thus prevent the occurrence of the serious AEs mentioned above (2-6). The standardisation of using glucose 5% as injection fluid instead of distilled water, which reduced vessel constriction/spasm and thus stasis, one of the causes for nontargeted microspheres disposition, also reduced the need for prophylactic embolization (7). Some studies showed that less vessel spasm was associated with reduced pain perception by the patient (8, 9), however our data shows that lack of prior coil or plug embolization can lead to an increase in AEBS, especially adverse events related to post-embolization syndrome. Finally, our cohort reported no occurrences of radiation pneumonitis, which is consistent with the generally low median lung shunt findings of 5-7% (10).

The occurrence of REILD should be a serious concern for interventional radiologists performing TARE treatments. REILD was first described in 2008 by Sangro et al. and is commonly understood as a combination of ascites, liver function derailment and elevated bilirubin levels 4-8 weeks after TARE treatment (11). Our study reported that 13/1027 patients (1.3%) experienced REILD, of which half were grade 3 or higher. This occurrence of REILD is on the lower end of the range reported in the systematic review of Braat et al., who identified that the incidence of symptomatic REILD varied between 0 and 31%, although, in most reports, the incidence was 0–8% (12). The same study summarised that previously found risk factors for REILD were liver-directed treatments prior to TARE, such as chemotherapy, external beam radiation therapy, other TARE treatments and the number of prior liver-directed therapies. Furthermore, administered activity per target volume or absorbed dose was identified as an independent risk factor, as well as single-session whole-liver treatments (13). The low occurrence of REILD in our cohort makes identifying predictive factors of REILD impossible but may indicate that treatment applications are adjusted to reduce the risk of REILD in patients treated with TARE. To prevent REILD, Sangro et al, 2017 recommended to avoid TARE for patients with poor liver function (bilirubin >2 mg/dL or non-tumoral ascites), and to consider reducing the activity for patients with chronic liver disease, (the liver is <1.5 L) or for patients who received multiple lines of chemotherapy prior to TARE (14).

Finally, post-embolization syndrome, characterised by abdominal pain, fatigue, nausea and vomiting, is a common occurrence after TARE (15) – in our cohort we found abdominal pain 13.9%, fatigue 11.4%, nausea 7.6% and vomiting 5.4% within 1 month after treatment. To reduce postradioembolization syndrome (nausea, fatigue, and pain), antiemetic and steroid medications can be given before the procedure, and patients are recommended to go home with a steroid taper, and antiemetic (as needed) (8). Since these symptoms are mild other recommendations suggest non-narcotic analgesics and ondansetron (14).

Quality of life

This prospective study shows that TARE has a minimum effect on QOL. Predictors for a deterioration of GHS were ECOG 0, >1 TARE treatment and partition model dosimetry. Previous studies have shown that patients treated with TARE for HCC maintain a stable quality of life outcome (16-18) and TARE is considered favourable to TACE in that respect (19-21). This is consistent with our findings, which shows no deterioration of GHS for HCC and ICC, and a minor deterioration of GHS after 6 months in the mCRC cohort. It should be noted that in mCRC, this deterioration is likely due to the development of the disease, as the PFS was only 3.4 months (22). Very few studies attempted to identify prognostic factors for deterioration of QOL. In Holmium-166 TARE, a higher World Health Organization performance score at baseline resulted in a decreased GHS (23). Our study furthermore found that there is a strong correlation between deterioration of GHS and an increase in AEBS, which may explain our results that partition model dosimetry and ECOG 0 predicted also a deterioration of GHS. Moreover, these patients were generally better off than their comparators, leading to improved effectiveness outcomes but also more time to show deterioration of the performance status compared to patients with a higher ECOG performance status (22). However, these results should be considered carefully, since only 43.9% of the patients completed the minimum required number of questionnaires: one at baseline questionnaire and at least one follow-up questionnaire.

**References**

1. Mahnken AH. Current status of transarterial radioembolization. World J Radiol. 2016;8(5):449-59.

2. Carretero C, Munoz-Navas M, Betes M, Angos R, Subtil JC, Fernandez-Urien I, et al. Gastroduodenal injury after radioembolization of hepatic tumors. Am J Gastroenterol. 2007;102(6):1216-20.

3. Ibrahim SM, Lewandowski RJ, Sato KT, Gates VL, Kulik L, Mulcahy MF, et al. Radioembolization for the treatment of unresectable hepatocellular carcinoma: a clinical review. World J Gastroenterol. 2008;14(11):1664-9.

4. Murthy R, Brown DB, Salem R, Meranze SG, Coldwell DM, Krishnan S, et al. Gastrointestinal complications associated with hepatic arterial Yttrium-90 microsphere therapy. J Vasc Interv Radiol. 2007;18(4):553-61; quiz 62.

5. Salem R, Thurston KG. Radioembolization with 90Yttrium microspheres: a state-of-the-art brachytherapy treatment for primary and secondary liver malignancies. Part 1: Technical and methodologic considerations. J Vasc Interv Radiol. 2006;17(8):1251-78.

6. Lam M, Banerjee S, Louie JD, Abdelmaksoud MHK, Iagaru AH, Ennen RE, et al. Root cause analysis of gastroduodenal ulceration after yttrium-90 radioembolization. Cardiovasc Intervent Radiol. 2013;36(6):1536-47.

7. Paprottka KJ, Lehner S, Fendler WP, Ilhan H, Rominger A, Sommer W, et al. Reduced Periprocedural Analgesia After Replacement of Water for Injection with Glucose 5% Solution as the Infusion Medium for 90Y-Resin Microspheres. J Nucl Med. 2016;57(11):1679-84.

8. Boas FE, Bodei L, Sofocleous CT. Radioembolization of Colorectal Liver Metastases: Indications, Technique, and Outcomes. J Nucl Med. 2017;58(Suppl 2):104s-11s.

9. Bilbao JI, Garrastachu P, Herraiz MJ, Rodriguez M, Inarrairaegui M, Rodriguez J, et al. Safety and efficacy assessment of flow redistribution by occlusion of intrahepatic vessels prior to radioembolization in the treatment of liver tumors. Cardiovasc Intervent Radiol. 2010;33(3):523-31.

10. Salem R, Parikh P, Atassi B, Lewandowski RJ, Ryu RK, Sato KT, et al. Incidence of radiation pneumonitis after hepatic intra-arterial radiotherapy with yttrium-90 microspheres assuming uniform lung distribution. Am J Clin Oncol. 2008;31(5):431-8.

11. Sangro B, Gil-Alzugaray B, Rodriguez J, Sola I, Martinez-Cuesta A, Viudez A, et al. Liver disease induced by radioembolization of liver tumors: description and possible risk factors. Cancer. 2008;112(7):1538-46.

12. Braat MN, van Erpecum KJ, Zonnenberg BA, van den Bosch MA, Lam MG. Radioembolization-induced liver disease: a systematic review. Eur J Gastroenterol Hepatol. 2017;29(2):144-52.

13. Gil-Alzugaray B, Chopitea A, Inarrairaegui M, Bilbao JI, Rodriguez-Fraile M, Rodriguez J, et al. Prognostic factors and prevention of radioembolization-induced liver disease. Hepatology. 2013;57(3):1078-87.

14. Sangro B, Martinez-Urbistondo D, Bester L, Bilbao JI, Coldwell DM, Flamen P, et al. Prevention and treatment of complications of selective internal radiation therapy: Expert guidance and systematic review. Hepatology. 2017;66(3):969-82.

15. Riaz A, Awais R, Salem R. Side effects of yttrium-90 radioembolization. Front Oncol. 2014;4:198.

16. Loffroy R, Ronot M, Greget M, Bouvier A, Mastier C, Sengel C, et al. Short-term Safety and Quality of Life Outcomes Following Radioembolization in Primary and Secondary Liver Tumours: a Multi-centre Analysis of 200 Patients in France. Cardiovasc Intervent Radiol. 2021;44(1):36-49.

17. Salem R, Hassan S, Lewandowski RJ, Grace K, Martin RCG, Sichlau MJ, et al. Quality of Life after Radioembolization for Hepatocellular Carcinoma Using a Digital Patient-Reported Outcome Tool. J Vasc Interv Radiol. 2020;31(2):311-4 e1.

18. Xing M, Kokabi N, Camacho JC, Kim HS. Prospective longitudinal quality of life and survival outcomes in patients with advanced infiltrative hepatocellular carcinoma and portal vein thrombosis treated with Yttrium-90 radioembolization. BMC Cancer. 2018;18(1):75.

19. Das A, Gabr A, O'Brian DP, Riaz A, Desai K, Thornburg B, et al. Contemporary Systematic Review of Health-Related Quality of Life Outcomes in Locoregional Therapies for Hepatocellular Carcinoma. J Vasc Interv Radiol. 2019;30(12):1924-33 e2.

20. Kirchner T, Marquardt S, Werncke T, Kirstein MM, Brunkhorst T, Wacker F, et al. Comparison of health-related quality of life after transarterial chemoembolization and transarterial radioembolization in patients with unresectable hepatocellular carcinoma. Abdom Radiol (NY). 2019;44(4):1554-61.

21. Salem R, Gilbertsen M, Butt Z, Memon K, Vouche M, Hickey R, et al. Increased quality of life among hepatocellular carcinoma patients treated with radioembolization, compared with chemoembolization. Clin Gastroenterol Hepatol. 2013;11(10):1358-65 e1.

22. Schaefer N, Grozinger G, Pech M, Pfammatter T, Soydal C, Arnold D, et al. Prognostic Factors for Effectiveness Outcomes After Transarterial Radioembolization in Metastatic Colorectal Cancer: Results From the Multicentre Observational Study CIRT. Clin Colorectal Cancer. 2022.

23. van Roekel C, Smits MLJ, Prince JF, Bruijnen RCG, van den Bosch M, Lam M. Quality of life in patients with liver tumors treated with holmium-166 radioembolization. Clin Exp Metastasis. 2020;37(1):95-105.
